# A highly conserved, inhibitable astacin metalloprotease from *Teladorsagia circumcincta* is required for cuticle formation and nematode development^☆^

**DOI:** 10.1016/j.ijpara.2015.01.004

**Published:** 2015-04

**Authors:** Gillian Stepek, Gillian McCormack, Alan D. Winter, Antony P. Page

**Affiliations:** Institute of Biodiversity, Animal Health & Comparative Medicine, University of Glasgow, Bearsden Road, Glasgow G61 1QH, UK

**Keywords:** *Caenorhabditis elegans*, *Teladorsagia circumcincta*, *Haemonchus contortus*, Astacin metalloprotease, Cuticle, Anthelmintic

## Abstract

Parasitic nematodes cause chronic, debilitating infections in both livestock and humans worldwide, and many have developed multiple resistance to the currently available anthelmintics. The protective collagenous cuticle of these parasites is required for nematode survival and its synthesis has been studied extensively in the free-living nematode, *Caenorhabditis elegans*. The collagen synthesis pathway is a complex, multi-step process involving numerous key enzymes, including the astacin metalloproteases. Nematode astacinsare crucial for *C. elegans* development, having specific roles in hatching, moulting and cuticle synthesis. NAS-35 (also called DPY-31) is a homologue of a vertebrate procollagen C-proteinase and performs a central role in cuticle formation of *C. elegans* as its mutation causes temperature-sensitive lethality and cuticle defects. The characterisation of DPY-31 from the ovine gastrointestinal nematode *Teladorsagia circumcincta* and its ability to rescue the *C. elegans* mutant is described. Compounds with a hydroxamate functional group have previously been shown to be potent inhibitors of procollagen C-proteinases and were therefore examined for inhibitory activity against the *T. circumcincta* enzyme. Phenotypic screening against *T. circumcincta*, *Haemonchus contortus* and *C. elegans* larval stages identified compounds that caused body morphology phenotypes consistent with the inhibition of proteases involved in cuticle collagen synthesis. These compounds correspondingly inhibited the activity of recombinant *T. circumcincta* DPY-31, supporting the hypothesis that this enzyme may represent a potentially novel anthelmintic drug target.

## Highlights

• Astacin metalloprotease, DPY-31, is conserved throughout the nematode phylum.
• DPY-31 is crucial to *Teladorsagia circumcincta* cuticle formation.
• Matrix metalloprotease inhibitors are efficacious against recombinant DPY-31.
• Novel hydroxamate inhibitors caused Dumpy and Moult defects in nematodes.
• DPY-31 is a potential target for future nematode control.

## Graphical abstract

**Figure:**
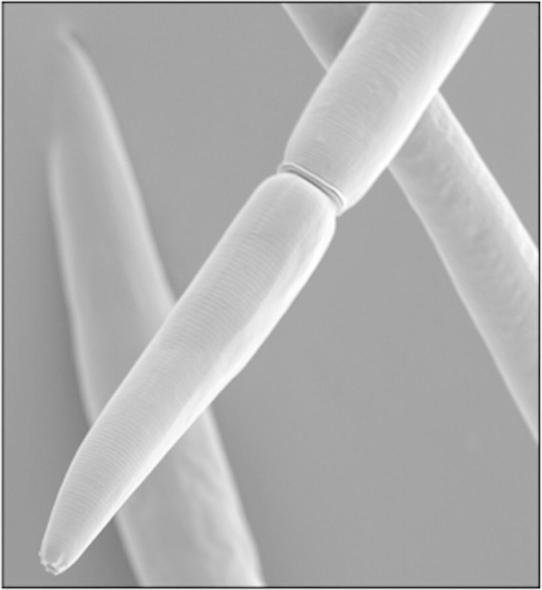

## 1 Introduction

Gastrointestinal (GI) nematodes cause chronic debilitating infections in livestock and humans worldwide, having a major economic impact on sheep farming resulting in loss of appetite, weight loss, decreased wool, meat and milk production and death (Zajac, 2006; Roeber et al., 2013). Current treatment is through the use of anthelmintic drugs (McKellar and Jackson, 2004); however, multiple resistance to anthelmintics of the three major classes has now developed in the veterinary parasites (Pomroy, 2006; Papadopoulos et al., 2012). Only a limited number of new drugs with novel modes of action have become available in recent years (Besier, 2007; Epe and Kaminsky, 2013), thereby limiting future prospects for effective control. No vaccines have yet been developed against these infections, although many different molecules have been under investigation for many years as potential vaccine candidates (Dalton and Mulcahy, 2001; Diemert et al., 2008; LeJambre et al., 2008).

All nematodes are surrounded by an external protective structure called the cuticle. The cuticle functions as an exoskeleton and provides protection from the external environment during development, hence its importance for nematode survival (Page et al., 2014). Synthesis of this structure is a complex, multi-step process, involving numerous enzymes (Page and Winter, 2003). The cuticle is largely composed of collagens (Fetterer, 1989; Johnstone, 2000), which are homologous between the free-living nematode, *Caenorhabditis elegans*, and parasitic nematodes such as the major GI nematodes of sheep, *Teladorsagia circumcincta* (Johnstone et al., 1996) and *Haemonchus contortus* (Laing et al., 2013). The process of cuticle biosynthesis has been studied in detail in *C. elegans* (Page and Winter, 2003), with many of the crucial cuticle synthesising enzymes and proteases also present in parasitic nematodes (reviewed in Page et al., 2014), suggesting that the cuticle biosynthesis process may be similar between *C. elegans* and its parasitic counterparts.

Protease enzymes are essential for the continued development and survival of nematodes in the host and fall into the following main classes: aspartic, cysteine, metallo-, threonine and serine proteases. The astacin metalloprotease enzymes play an essential role in cuticle biosynthesis in *C. elegans* (Hishida et al., 1996; Davis et al., 2004; Novelli et al., 2004, 2006; Suzuki et al., 2004). These enzymes are structurally distinct zinc metallo-endopeptidases that are characterised by two conserved motifs in the N-terminal astacin domain: the zinc-binding active site (HExxHxxGFxHExxRxDRD) and the methionine-turn (SxMHY) (Bond and Beynon, 1995). Binding of the zinc in the active site is essential for the catalytic activity of the enzyme; this zinc is pentacoordinated in a trigonal–bipyramidal geometry between the three histidine residues in the binding motif, the tyrosine in the methionine-turn and a water molecule (Bode et al., 1992). The first astacin metalloprotease identified was found in the crayfish, *Astacus astacus*, and consisted of a signal peptide, prodomain and a catalytic protease domain containing both conserved catalytic motifs. Astacin metalloproteases containing C-terminal domains of unknown functions, as well as the N-terminal catalytic domain, have now been isolated from a range of organisms including humans, mice, *Drosophila melanogaster* and *C. elegans* (Stöcker et al., 1993; Möhrlen et al., 2003, 2006). The primary role in all species is in development (Bond and Beynon, 1995), such as the hatching and moulting of *C. elegans* (Hishida et al., 1996; Davis et al., 2004; Suzuki et al., 2004). Functional roles for astacin proteases in parasitic nematodes include host tissue penetration by infective L3s (Williamson et al., 2006), cuticle formation and ecdysis (Gamble et al., 1989; Stepek et al., 2010, 2011) and digestion (Gallego et al., 2005).

There are 39 nematode astacin (NAS) metalloproteases expressed in *C. elegans*, which are subdivided into six subgroups (I to VI). Based on expression studies and RNA interference (RNAi), many of these proteases play developmental roles in *C. elegans* (Möhrlen et al., 2003). All the *C. elegans* NAS have a similar domain arrangement: signal peptide, prodomain, N-terminal catalytic astacin domain and may include a combination of the following C-terminal domains: Epidermal Growth Factor (EGF), Complement component Uegf and BMP-1 (CUB) and ThromboSPondin type-1 repeat (TSP-1) (Möhrlen et al., 2003). Removal of the prodomain causes conformational changes to the astacin domain, which results in enzyme activation (Guevara et al., 2010). The functions of the C-terminal domains are largely unknown but these domains, whilst having a non-catalytic purpose, are hypothesised to regulate the catalytic activity of the enzyme, provide its specificity and determine when and where the protein performs its role (Wermter et al., 2007). The subgroup V enzymes, NAS-33 to NAS-38, are found only in nematodes and have a unique nematode-specific C-terminal domain arrangement, consisting of one EGF, one CUB and one TSP-1 domain (Möhrlen et al., 2003). The enzyme NAS-34 is required for embryo hatching (Hishida et al., 1996) and NAS-36 and NAS-37 are both crucial to the moulting process (Davis et al., 2004).

DPY-31 (also known as NAS-35) has similarities to the vertebrate procollagen C-proteinase Bone Morphogenetic Protein-1 (BMP-1), which is critical for the assembly of collagen fibres during cartilage and bone formation through its excision of the C-terminal domain of procollagen to form mature collagen (Li et al., 1996). In *C. elegans*, *dpy-31* is expressed throughout the life-cycle, particularly in the embryonic and larval stages, in most hypodermal cells, as well as the rectal and vulval epithelial cells (Novelli et al., 2004). *Caenorhabditis elegans dpy-31(e2770)* mutants are inviable at the standard growth temperature of 20 °C and partially viable at 15 °C, with survivors displaying severe body morphology defects known as Dumpy (short and fat) (Novelli et al., 2004). Genetic suppressor screens identified the DPY-31 cleavage site in the C-terminus of the essential cuticle collagen SQT-3 (Novelli et al., 2006). The procollagens, such as SQT-3, in *dpy-31(e2770)* mutants therefore remain partially processed and cannot form mature collagens. Thus, DPY-31 plays a crucial role in cuticle formation and the moulting process in *C. elegans*. Earlier work has indicated that hydroxamate-based compounds are highly effective inhibitors of procollagen C-proteinase (Ovens et al., 2000; Fish et al., 2007; Bailey et al., 2008), suggesting that these compounds may also be effective against the nematode DPY-31 enzymes.

Here we identify and characterise the DPY-31 orthologue from the important GI nematode of sheep, *T. circumcincta*. The naturally occurring hydroxamic acid, actinonin (Gordon et al., 1975), and other developed inhibitory compounds containing a hydroxamate functional group (Fish et al., 2007) were screened against recombinant *T. circumcincta* DPY-31 and against larval stages of *T. circumcincta*, *H. contortus* and *C. elegans* to determine their effectiveness as inhibitors of this key developmental enzyme.

## 2 Materials and methods

### 2.1 Nematode strains and culture

*Caenorhabditis elegans* was cultured as described previously (Stiernagle, 2006), with the strains N2 (wild-type), HT1593 (*unc-119*(*ed3*) III) and DR96 (*unc-76*(*e911*) V) provided by the *Caenorhabditis* Genetics Center (CGC), USA. The *C. elegans dpy-31(e2770)* strain was provided by Professor Jonathan Hodgkin, University of Oxford, UK.

*Teladorsagia circumcincta* MTci2 and *H. contortus* ISE strains were provided by Professor David Knox (Moredun Research Institute, UK). Nematode eggs were extracted from infected sheep faeces using a salt flotation protocol and L1s were allowed to hatch in tap water at approximately 25 °C overnight. These L1s were cleaned free from eggs and faecal matter by passing down a Baermann apparatus overnight. Active, healthy *T. circumcincta* L3s (<6 months old) were exsheathed by exposure to CO_2_, as detailed in Halliday et al. (2012), except that the larvae were initially activated at 40 °C in a New Brunswick Scientific, USA (E24 Series) shaker at 60 rpm for 15 min, exposed to CO_2_ in a total volume of 5 ml, and then incubated at 40 °C with shaking at 100 rpm for 90 min. The exsheathment progress of a 20 μl sample was monitored under a Zeiss dissecting microscope.

### 2.2 Preparation of genomic DNA, RNA and cDNA

Genomic DNA was isolated from adult *T. circumcincta* (MTci5) by homogenisation in Proteinase K and repeated phenol:chloroform extractions, as described previously (Stepek et al., 2010). For RNA, adult nematodes, L3s or L1s were disrupted in TRIzol (Life Technologies, UK) using a hand-held Pestle and Motor Mixer (VWR, UK) with 1.5 ml disposable pestles and screw-capped tubes (VWR), and total RNA isolated following the TRIzol (Life Technologies) protocol. cDNA was prepared using an AffinityScript Multiple Temperature cDNA synthesis kit (Agilent Technologies, USA).

### 2.3 Identification of the coding and genomic sequences of *T. circumcincta dpy-31*

The *C. elegans* DPY-31 protein sequence was used to tBLASTn search the *T. circumcincta* database (M. Mitreva, Washington University School of Medicine, USA, personal communication). The supercontig0014679 and scaffold00169 had the highest homology score, and potential intron–exon splice sites were predicted using GeneWise2 (http://www.ebi.ac.uk/Tools/psa/genewise/). The translated sequence was aligned with the *C. elegans* protein sequence using ClustalX and BoxShade (http://www.ch.embnet.org/software/BOX_form.html). Amplification of the 5′ and 3′ ends of the *T. circumcincta dpy-31* gene was performed using the 5′ and 3′ RACE Systems for Rapid Amplification of cDNA Ends (Life Technologies) with the primers dpy-315′1, dpy-315′2 and dpy-31a3′ (Supplementary Table S1). Gene Structure Draw (http://www.compgen.uni-muenster.de/tools/strdraw/?bscl=false&lang=en&mscl=0&cscl=0) was used to produce a scaled schematic depicting the positions of the introns and exons.

DPY-31 orthologues from across the nematode phylum were identified using BLASTp with *C. elegans* DPY-31 as a query in WormBase (http://www.wormbase.org/tools/blast_blat), the Wellcome Trust Sanger Institute, UK (http://www.sanger.ac.uk/resources/software/blast/) and GenBank (http://www.ncbi.nlm.nih.gov/BLAST/Blast.cgi?PROGRAM=blastp&PAGE_TYPE=BlastSearch&LINK_LOC=blasthome). These sequences were aligned using ClustalX2 and a phylogenetic tree generated using ClustalW2 – Phylogeny (http://www.ebi.ac.uk/Tools/phylogeny/clustalw2_phylogeny/) and Phylodendron (Phylogenetic tree printer; http://iubio.bio.indiana.edu/treeapp/treeprint-form.html).

### 2.4 Generation of integrated transgenic *C. elegans* lines with a *T. circumcincta dpy-31* rescue plasmid using microparticle bombardment

Plasmid p*nas-35*, containing the *C. elegans dpy-31* promoter and 3′ untranslated region (UTR), was constructed as described previously (Stepek et al., 2010). The coding sequence of the *T. circumcincta dpy-31* gene was amplified from *T. circumcincta* cDNA by PCR using the primers Tcdpy-31SbfIF and Tc35aR (Supplementary Table S1) and *PfuUltra* polymerase. The 1845 bp product was cloned into plasmid pCR-TOPO2.1 (Life Technologies), a synthetic intron inserted by ligation of a double-stranded oligonucleotide (5′-gtaagtttaaactattcgttactaactaactttaaacatttaaattttcag-3′) into a *Pml*I restriction site, and then subcloned into p*nas-35* using *Sbf*I and *Not*I sites to create the *T. circumcincta dpy-31* rescue construct, named *Tcdpy-31* + SI/p*nas-35*. This plasmid was sequenced over junctions using the primers Ce35pinF, Ce35uinR and Tcdpy-31SIseqF (Supplementary Table S1).

Transgenic *C. elegans* lines were generated with the plasmid *Tcdpy-31* + SI/p*nas-35* using a microparticle bombardment method essentially as described (Praitis, 2006). Briefly, the *C. elegans* mutant strain HT1593 (*unc-119(ed3)*) was grown in liquid culture using *Escherichia coli* strain HB101 (Praitis, 2006; Stiernagle, 2006), and microparticle bombardment was performed using the biolistic PDS-100/He particle delivery system with a Hepta Adaptor, using 5 μg of *Tcdpy-31* + SI/p*nas-35* and 5 μg of pDPMM016b (*unc-119*(+), rescue plasmid) (Maduro and Pilgrim, 1995). The bombarded worms were incubated at 25 °C for >2 weeks, after which non-Unc dauers were cloned. Three integrated strains, TP204, TP205 and TP207, were isolated and the presence of the parasite gene confirmed by single-worm PCR (Williams et al., 1992) with the primers Tcdpy-31SbfI F and Tc35aR. Strain TP224 (*dpy-31(e2770)* III; kaIs8[*Tcdpy-31* + SI/p*nas-35* + *unc-119*(+)]) was created by crossing TP205 (*Tcdpy-31* + SI/p*nas-35* + *unc-119*(+)) and *dpy-31(e2770)* at 15 °C. The transgene was followed through crosses by single-worm PCR (primers as above) and the *dpy-31(e2770)* allele followed using allele-specific PCR (Gaudet et al., 2009) with the primers *dpy-31*F, *dpy-31*(M)F and *dpy-31*R2 (Supplementary Table S1). Homozygosity of the transgene was demonstrated by transmission to 100% of progeny tested (*n* = 20). To confirm the function of *T. circumcincta dpy-31* in the strain TP224, the *T. circumcincta dpy-31* RNAi plasmid (*Tcdpy-31* insert subcloned into the RNAi vector, L4440) was transformed into bacterial strain HT115(DE3) and RNAi feeding was performed on the N2 and TP224 strains at 20 °C. HT115(DE3) cells containing the RNAi vector without an insert was used as a negative control.

### 2.5 Generation of transgenic *C. elegans* lines with a *T. circumcincta dpy-31* promoter–reporter plasmid by microinjection

A 1626 bp fragment was amplified from *T. circumcincta* genomic DNA (positions −1617 to +9 relative to the *dpy-31* ATG start) using primers Tcdpy-31repF2 and Tcdpy-31repR2 (Supplementary Table S1), cloned into the reporter gene vector pPD96:04 (*lac*Z::*gfp*; Addgene) using *Sma*I and *Sal*I restriction sites, and sequenced using the primers Reporter seq and M13Rev(-29) (Supplementary Table S1). This construct was microinjected into the syncytial gonad of *C. elegans* strain DR96 (*unc-76*(*e911*) V) at 100 μg/ml together with p7616B (*unc-76*(+), rescue plasmid) at 100 μg/ml. Three transgenic lines were identified and examined for reporter gene expression by staining glutaraldehyde-fixed worms for β-galactosidase activity (Hope, 1991).

### 2.6 Recombinant expression of *T. circumcincta* DPY-31

A 684 bp synthetic gene encoding the astacin domain of *T. circumcincta* DPY-31, with codons optimised for *E. coli*, was generated by GeneArt (Life Technologies, UK) and cloned into the pET-28a(+) vector (Novagen, Germany) using *Nde*I and *Xho*I. This construct was fully sequenced, transformed into *E. coli* BL21 (DE3) cells, and recombinant protein expression induced with 0.5 mM isopropyl β-d-1-thiogalactopyranoside (IPTG) at 16 °C, 250 rpm overnight. Cells were harvested and stored at −80 °C then resuspended in native lysis buffer, pH 8.0 (50 mM NaH_2_PO_4_, 300 mM NaCl, 10 mM imidazole), 1.7 mg/ml of lysozyme and 0.1 mg/ml of DNaseI. The lysed cells were sonicated and purification of the protein from the soluble cell lysate was performed using Ni-NTA resin columns (Qiagen, UK) under native conditions, with 20 mM imidazole in the wash buffer (50 mM NaH_2_PO_4_, 300 mM NaCl) and 250 mM imidazole in the elution buffer (50 mM NaH_2_PO_4_, 300 mM NaCl). The eluates were concentrated together using a 10 kDa cut-off concentrator (Millipore, Germany) and then analysed by SDS–PAGE and Western blotting using an anti-histidine (His) G antibody (Life Technologies, UK) and an anti-mouse IgG (whole molecule) alkaline phosphatase conjugate (Sigma, UK). Protein concentration was determined using the Bradford assay.

### 2.7 Zinc metalloprotease activity assays

Zinc metalloprotease assays were performed on recombinant *T. circumcincta* DPY-31 using an adapted method (Gamble et al., 1989, 1996; Stöcker and Zwilling, 1995). Briefly, 0, 25, 50, 100 or 200 μg/ml of recombinant enzyme were incubated with 0.1 mM ZnCl_2_, and the total volume was made up to 100 μl with 50 mM NaPO_4_, pH 8.0 buffer prior to the addition of 1 mM synthetic substrate, Suc-Ala-Ala-Ala-pNA (Bachem, Germany), and incubation at 37 °C for 3 h. Each sample was performed in duplicate and the absorbance was measured on a plate reader at 405 nm. A second assay involved incubating 150 μg/ml of recombinant enzyme with 0.1 mM ZnCl_2_ and 0, 25, 50, 100 or 200 μM inhibitor, 1,10-phenanthroline, with the volume made up to a total of 100 μl with 50 mM NaPO_4_, pH 8.0 buffer. Pre-incubation occurred for 3 h at 37 °C prior to the addition of 1 mM Suc-Ala-Ala-Ala-pNA and subsequent incubation at 37 °C for a further 3 h. Each sample was performed in duplicate and the absorbance was measured on a plate reader at 405 nm.

### 2.8 In vitro screening of compounds against recombinant *T. circumcincta* DPY-31

Actinonin and four further hydroxamate-based compounds that have exhibited procollagen C-proteinase inhibition (compounds 1, 25, 26 and 56 described in Fish et al. (2007)) were made up in 50–100% ethanol and tested in an astacin assay to determine their ability to inhibit recombinant *T. circumcincta* DPY-31. Briefly, 150 μg/ml of recombinant enzyme was incubated with 0.1 mM ZnCl_2_ and compound at concentrations ranging from 5 μM to 100 μM, and the volume was made up to a total of 100 μl with 50 mM NaPO_4_, pH 8.0 buffer. Pre-incubation occurred for 3 h at 37 °C prior to the addition of 1 mM Suc-Ala-Ala-Ala-pNA (Bachem) and subsequent incubation at 37 °C for a further 3 h. Absorbance was measured on a plate reader at 405 nm, with each sample performed in duplicate.

### 2.9 In vivo screening of compounds

The five compounds described in Section 2.8 were screened against *C. elegans* wild-type (N2) or *T. circumcincta*-rescued *dpy-31* mutants (strain TP224) using 96-well plates. *Caenorhabditis elegans* L4s were placed into each of the wells in a total volume of 100 μl, made up of compound, M9 buffer and 5 μl of 10× concentrated food (*E. coli* strain OP50). Each compound was tested in duplicate at concentrations ranging from 50 μM to 2 mM. The plates were sealed with gas-permeable covers and incubated at 20 °C for 3 days, with viability and gross morphology of the F1 progeny recorded daily. On day 3, representative offspring were transferred onto 2% agarose/0.01% azide pads on slides and viewed by microscopy.

### 2.10 Screening of compounds against *T. circumcincta* and *H. contortus* larvae

L1s or exsheathed L3s of *T. circumcincta* and *H. contortus* were incubated with the same compounds as in Section 2.9. Each compound was tested in duplicate at concentrations ranging from 50 μM to 2 mM for both L1s and exsheathed L3s. L1s were incubated, 10–20 per well of a 96-well plate, each well containing compound, 5 μl of 10× concentrated OP50 and Earles Balanced Salt Solution (EBSS, Life Technologies) to 100 μl; or 10–20 exsheathed L3s were incubated per well of a 96-well plate, each well containing compound and EBSS to 100 μl. The plates were sealed with gas-permeable covers and incubated at 26 °C (L1s) or 40 °C (L3s) at 100 rpm for 7–8 days and any observations on development were recorded. Representative worm images were taken as stated in Section 2.9.

### 2.11 Microscopy

Live nematodes, mounted on 2% agarose/0.01% azide pads, or fixed nematodes, were viewed under Differential Interference Contrast (DIC) or fluorescence (GFP) optics on a Zeiss Axioskop2 microscope, and images were taken using an AxioCam camera and Axiovision software.

## 3 Results

### 3.1 Identification of *T. circumcincta* dpy-31

**Fig. 1:**
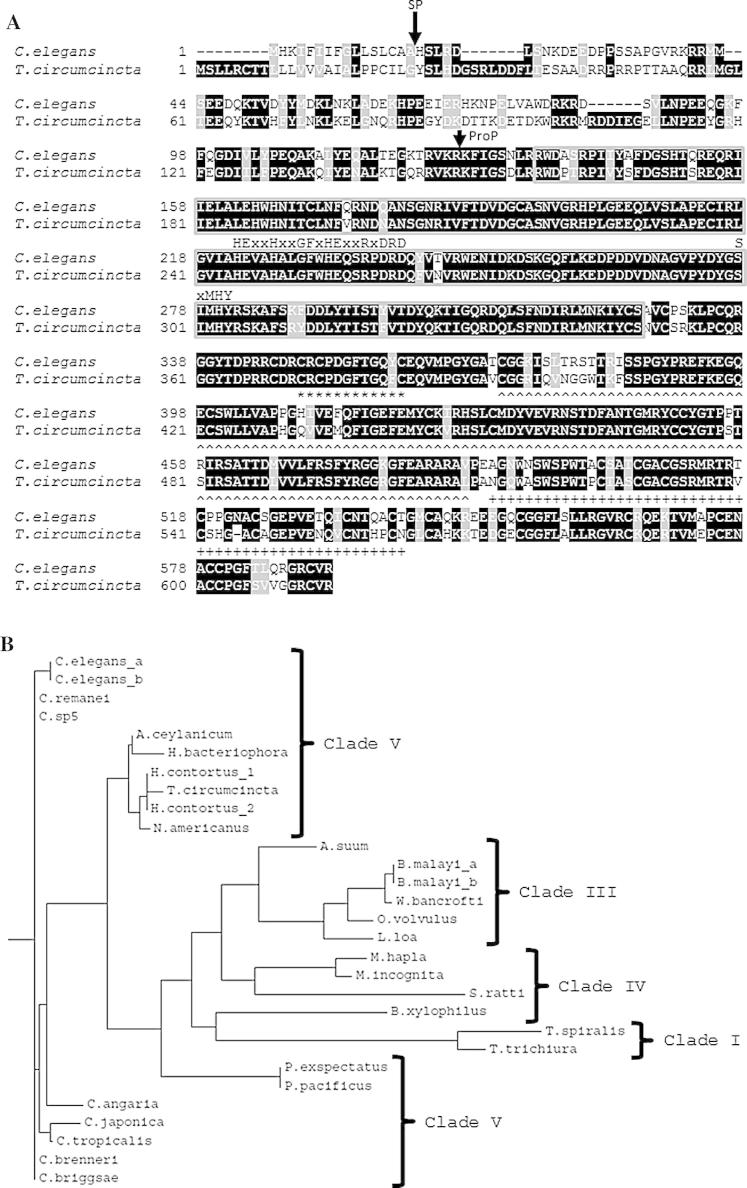
Comparison of nematode DPY-31 proteins. (A) Alignment of DPY-31 from *Caenorhabditis elegans* and *Teladorsagia circumcincta*. Alignment was performed in ClustalW and BoxShade, with identical amino acids shaded black and similar amino acids shaded grey. The signal peptide (SP) and prodomain (ProP) are indicated by arrows. The catalytic active site and conserved methionine turn are labelled with HExxHxxGFxHExxRxDRD and SxMHY above the sequence, respectively. The domains are highlighted as follows: grey box, astacin domain; ∗ below the sequence, Epidermal Growth Factor domain; ^ below the sequence, Complement component Uegf and BMP-1 domain; and + below the sequence, ThromboSPondin type-1 repeat domain. (B) Phylogenetic analysis of protein sequence data illustrating the relationship of DPY-31 between parasitic and free-living nematodes across phylogenetic clades, I, III, IV and V. DPY-31 sequences were obtained via BLASTp of databases at WormBase, the Wellcome Trust Sanger Institute, UK and GenBank, and were then aligned using ClustalX2 and arranged in a phylogenetic tree using ClustalW2 (Phylogeny) and Phylodendron. GenBank Accession Numbers: *C. elegans* isoform A (CCD70758), *C. elegans* isoform B (CCD70759), *Caenorhabditis brenneri* (EGT53190.1), *Caenorhabditis briggsae* (XP 002641922.1), *Caenorhabditis remanei* (XP 003112951.1), *Caenorhabditis japonica* (CJP-DPY-31), *Caenorhabditis angaria* (Cang_2012_03_13_00993.g15272.t2), *Caenorhabditis tropicalis* (Csp11.scaffold489.g2034.t1), *Caenorhabditis* sp5 (Csp5_scaffold_00350.g9906.t1), *T. circumcincta* (KM272923), *Ancylostoma ceylanicum* (EYC02674.1), *Haemonchus contortus* isoform 1 (CDJ87214.1), *H. contortus* isoform 2 (CDJ95996.1), *Heterorhabditis bacteriophora* (ABY74338.1), *Necator americanus* (ETN74486.1), *Pristionchus pacificus* (PPA-DPY-31), *Pristionchus exspectatus* (scaffold21_EXSNAP2012.38), *Ascaris suum* (ADY45142), *Brugia malayi* isoform A (ACZ64270.1), *B. malayi* isoform B (ACZ64271.1), *Wuchereria bancrofti* (EJW84352.1), *Loa loa* (EF026205), *Onchocerca volvulus* (OVO-DPY-31), *Trichinella spiralis* (EFV57669), *Trichuris trichiura* (CDW53272.1), *Strongyloides ratti* (CEF68555.1), *Meloidogyne hapla* (MhA1_contig704.frz3.gene1), *Meloidogyne incognita* (Minc01936) and *Bursaphelenchus xylophilus* (BUX.s00351.406).

Following BLAST searches in the *T. circumcincta* database, a *dpy-31* orthologue was identified and its sequence confirmed by 5′- and 3′-RACE and amplification of a full-length PCR product. Although two 3′ alternatively-spliced forms (A and B) of *dpy-31* have been isolated from *C. elegans*, only isoform A was found in *T. circumcincta*. The *T. circumcincta dpy-31* gene is 17,346 bp, consisting of 16 exons (data not shown) compared with the *C. elegans* gene, which is 5003 bp and eight exons. The full-length coding sequences for *dpy-31* in *T. circumcincta* and *C. elegans* are 1845 bp and 1779 bp, and encode polypeptides of 614 and 592 amino acids, respectively. Signal peptide cleavage sites and pro-domain cleavage sites for *T. circumcincta* DPY-31 were predicted using the SignalP and ProP programs (Fig. 1A). Thus, the predicted mature proteins for DPY-31 consist of 465 and 466 amino acids for *T. circumcincta* and *C. elegans*, respectively, having an identity of 85.4%. The size and pIs of the mature DPY-31 proteins, calculated using Compute pI/MW (http://web.expasy.org/compute_pi/), are 52.3 kDa and 52.6 kDa, and 7.71 and 8.16 for *T. circumcincta* and *C. elegans*, respectively. The DPY-31 proteins from both *T. circumcincta* and *C. elegans* have an N-terminal catalytic astacin domain (Pfam PF01400), containing the crucial zinc-binding site which is characterised by the sequence HExxHxxGFxHExxRxDRD, and a conserved methionine-turn (SxMHY), of which the tyrosine residue forms one of the five important ligands essential to bind the active-site zinc. In addition to the essential catalytic domain, the DPY-31 proteins also contain successive EGF, CUB and TSP-1 domains at the C-terminal (Fig. 1A).

A phylogenetic tree, shown in Fig. 1B, was generated with DPY-31 orthologues from nematode species across the phylum. Fig. 1B indicates that DPY-31 orthologues are largely organised into the different nematode clades, with identity to *C. elegans* DPY-31 ranging from 57% in Clade I to 100% in Clade V. The presence of DPY-31 orthologues throughout the nematode phylum supports a conserved, crucial role for this protease during nematode development.

### 3.2 Complementation of the *C. elegans dpy-31* mutant with the *T. circumcincta dpy-31* gene

**Fig. 2:**
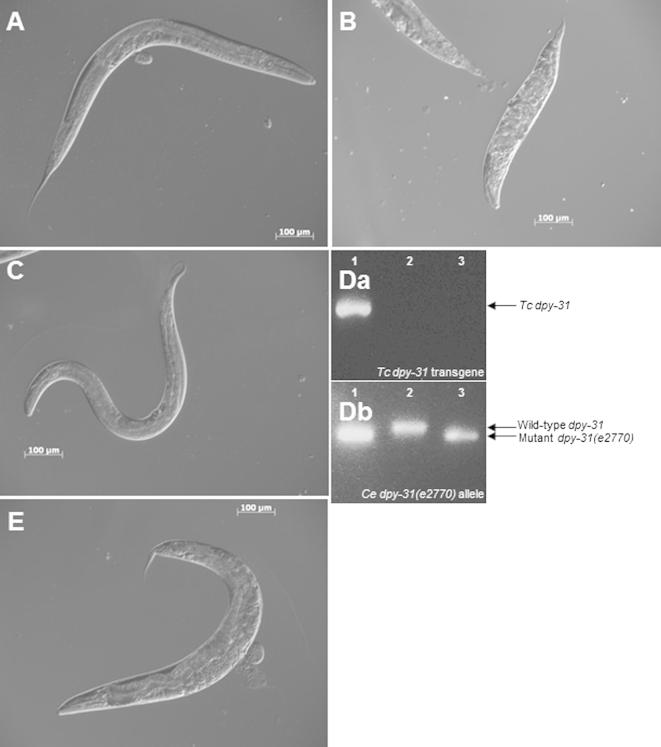
Complementation of a *Caenorhabditis elegans dpy-31(e2770)* mutant with *Teladorsagia circumcincta (Tc) dpy-31*. A *C. elegans (Ce) dpy-31(e2770)* mutant was crossed with an integrated transgenic line carrying the wild-type *Tc dpy-31* gene. (A) An adult N2 at 20 °C; (B) an adult *dpy-31(e2770)* mutant at 15 °C; and (C) an adult TP224 (*T. circumcincta*-rescued *dpy-31(e2770)* mutant) at 20 °C. (D) A single-worm allele-specific PCR is shown to demonstrate the presence of the *Tc dpy-31* gene and *Ce dpy-31(e2770)* mutant allele in TP224 (lane 1, representative TP224 F3; lane 2, adult N2; and lane 3, adult *dpy-31(e2770)*). (a) The presence of the *Tc dpy-31* gene in the TP224 transgenic strain, but not in N2 wild-type or *dpy-31(e2770)* mutant strains is shown. (b) The *dpy-31(e2770)* allele is a homozygous mutant in both the transgenic TP224 strain and the original *dpy-31(e2770)* mutant, but wild-type in the N2 strain. (E) *Tc dpy-31* RNA interference by feeding the rescued TP224 wild-type transgenic strain reverts back to the mutant phenotype.

The complex lifecycles of parasitic nematodes, such as *T. circumcincta,* make them less amenable to experimental analysis. To overcome these difficulties, *C. elegans* was applied as a surrogate experimental system. The *C. elegans dpy-31(e2770)* mutant is a temperature-sensitive larval lethal strain that is non-viable at 20 °C and barely viable at 15 °C, with all surviving worms exhibiting a strong recessive body morphology phenotype known as Dumpy (Novelli et al., 2004). Introduction of *T. circumcincta dpy-31* into the *C. elegans dpy-31(e2770)* strain fully repaired the lethality and body morphology phenotypes of this mutant. Rescued strains were confirmed as homozygous for mutation in endogenous *C. elegans dpy-31* by allele-specific single-worm PCR. Fig. 2A shows a wild-type *C. elegans* N2, while Fig. 2B shows the Dumpy phenotype of the *dpy-31(e2770)* mutant at 15 °C. These images were compared with Fig. 2C to indicate that the *T. circumcincta dpy-31* gene was able to rescue the *dpy-31(e2770)* mutant at the normally non-permissive temperature of 20 °C. Fig. 2D shows single-worm PCR of representative rescued worms (TP224) and compares them with the original mutant *dpy-31(e2770)* and wild-type (N2) backgrounds. Primers were able to detect the presence of the *T. circumcincta dpy-31* transgene in 100% of the progeny tested, but not in either the N2 or *dpy-31(e2770)* strains (Fig. 2Da); allele-specific genotyping primers indicated that the *dpy-31(e2770)* allele is a homozygous mutant in both TP224 and the original *dpy-31(e2770)* mutant, but wild-type in N2 (Fig. 2Db). Confirmation that the *T. circumcincta dpy-31* gene was required for the complementation of the TP224 strain was provided when this strain was subjected to *T. circumcincta dpy-31* RNAi, with the resultant progeny returning to a mutant Dumpy phenotype with a penetrance ranging from 60% to 100% (Fig. 2E). This supports the hypothesis that the *dpy-31* gene in *T. circumcincta* shares the same important function as *dpy-31* in *C. elegans*.

### 3.3 Heterologous expression of the *T. circumcincta dpy-31* promoter–reporter in *C. elegans*

**Fig. 3:**
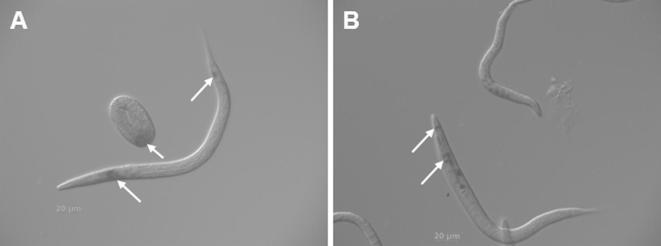
Promoter–reporter expression of *Teladorsagia circumcincta* DPY-31 in *Caenorhabditis elegans*. Approximately 2 kb of the *T. circumcincta dpy-31* promoter was cloned into the reporter vector, pPD96:04, and *C. elegans* transgenic lines established. Following β-galactosidase staining, expression was apparent in three independent lines, and throughout the life-cycle stages. Representative images of larvae and embryos are depicted in (A) and (B). The arrows indicate expression in the pharyngeal gland cells and rectal epithelial cells.

A *T. circumcincta dpy-31* promoter–reporter construct was used to examine tissue-specific localisation by its ability to direct spatial expression in *C. elegans*. Detectable transgene expression is predominantly restricted to the pharyngeal gland cells and the rectal epithelial cells of *C. elegans*, as demonstrated by β-galactosidase staining of transgenic *C. elegans* (Fig. 3). This expression pattern was consistent throughout all life-cycle stages, in particular embryos and larvae (Fig. 3), and is consistent with a nematode enzyme that is expressed in the posterior gut and the glandular excretory/secretory system.

### 3.4 Recombinant expression of *T. circumcincta* DPY-31

**Fig. 4:**
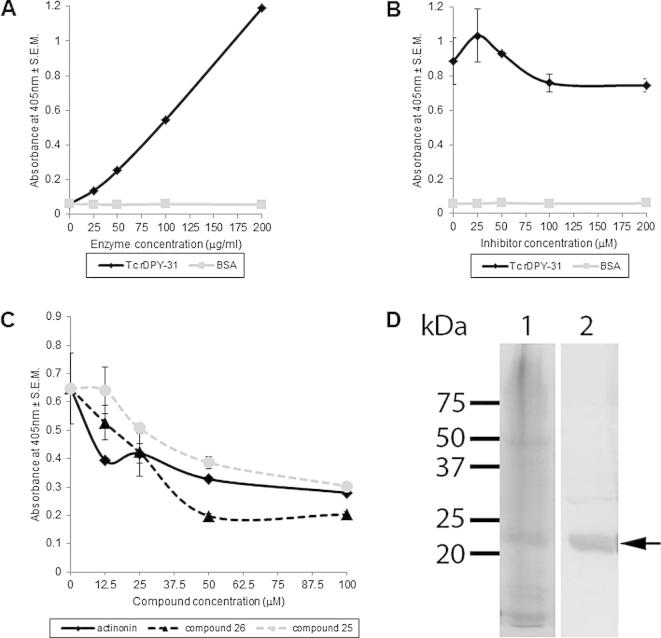
Metalloprotease activity of recombinant DPY-31 from *Teladorsagia circumcincta* in the presence and absence of inhibitory compounds. (A) An increase in absorbance is shown as the concentration of recombinant protein also increases from 25 to 200 μg/ml. (B) The progressive inhibition of recombinant Tc DPY-31 (150 μg/ml) metalloprotease activity in the presence of increasing concentrations (25–200 μM) of the general metalloprotease inhibitor, 1,10-phenanthroline is shown. (C) The effect on metalloprotease activity of 150 μg/ml of recombinant protein on incubation with increasing concentrations of inhibitory hydroxamate-containing compounds 25 and 26 is shown. (D) Coomassie staining of histidine (His) column-eluted recombinant Tc DPY-31 (lane 1) and an anti-His tag Western blot of eluted recombinant Tc DPY-31 (lane 2). Arrow indicates recombinant protein.

The *T. circumcincta* DPY-31 catalytic astacin domain was cloned into the pET-28a(+) vector for expression in *E. coli*, and the His-tagged recombinant protein induced using IPTG. The corresponding Ni-NTA column-purified 23 kDa protein was detected by Western blotting following probing with an anti-His antibody (Fig. 4D). The resulting purified recombinant protein was found to be active in an astacin activity assay against a synthetic peptide substrate (Fig. 4A). Metalloprotease activity was zinc-dependent and was weakly but progressively inhibited by the general zinc metalloprotease inhibitor, 1,10-phenanthroline (Fig. 4B). This result confirms that DPY-31 from the parasitic nematode, *T. circumcincta*, is indeed a specific, functionally-active, zinc metalloprotease.

### 3.5 Screening of compounds with potential to inhibit nematode astacin metalloproteases

Actinonin and procollagen metalloprotease inhibitors 1, 25, 26 and 56 (Fish et al., 2007) have proven to be effective, reversible inhibitors of astacins and procollagen C-peptidases. These five compounds were screened against the recombinant *T. circumcincta* DPY-31 protein (Table 1) and, as can be seen in Fig. 4C, compound 26 was the most efficacious, displaying more potent inhibition than the hydroxamate-based inhibitor, actinonin. Compounds 25, 26 and actinonin had a much greater inhibitory activity against recombinant DPY-31 than the generic metalloprotease inhibitor, 1,10-phenanthroline (Fig. 4B, C), suggesting that recombinant DPY-31 has greater binding specificity to compounds with a hydroxamate functional group than to those without this functional group. The related hydroxamate compounds 1 and 56, however, consistently proved to be ineffective at inhibiting the activity of recombinant *T. circumcincta* DPY-31 (Table 1). This indicates, that in addition to the hydroxamate structure, adjacent functional groups may play a role in the specificity.

**Fig. 5:**
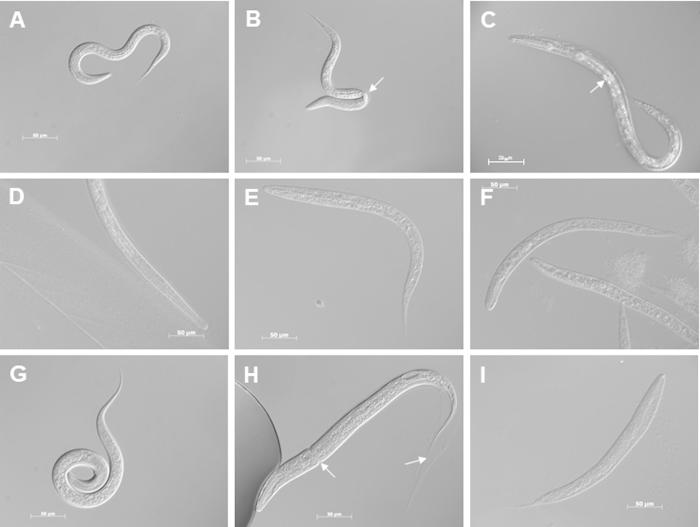
Effect of metalloprotease inhibitors on the development of *Caenorhabditis elegans*, *Teladorsagia circumcincta* and *Haemonchus contortus* larvae. (A–C) *Caenorhabditis elegans*; (D–F) *T. circumcincta*; (G–I) *H. contortus*. (A) A wild-type L2 offspring in the absence of compound at 3 days is shown, and (D and G) show wild-type L3s in the absence of compound at 7 days. (B) A L2 offspring with a Moult (Mlt) defect on incubation with 100 μM actinonin for 3 days is shown. (E) A L3 with a mild Dumpy (Dpy) defect on incubation with 500 μM actinonin for 7 days, and (H) a L3 with a Mlt defect on incubation with 200 μM actinonin for 7 days. (C) A L4 offspring with a Mlt defect on incubation with 300 μM compound 25 for 3 days, (F) a L3 with a mild Dumpy phenotype on incubation with 100 μM compound 25 for 7 days, and (I) a L3 with a strong Dumpy phenotype on incubation with 500 μM compound 25 for 7 days. Mlt defects in (B, C and H) are indicated by white arrows.

When these same five compounds were screened against the L4s of *C. elegans* wild-type (N2) and *T. circumcincta*-rescued *dpy-31(e2770)* mutants (*C. elegans* strain TP224), Dumpy worms and cuticle defects (Moult) were observed (Table 1; Fig. 5A–C). These effects were similar for both of the *C. elegans* strains being tested, with the weakest effects being observed with compounds 1 and 56, and being consistent with their poor inhibition of the recombinant enzymes. When compounds 25 and actinonin were screened against the L1 and exsheathed L3 of *T. circumcincta* and against the L1 of *H. contortus*, these worms became immobile and had a sick appearance. These nematodes also showed a range of Moult (Fig. 5H) and Dumpy (Fig. 5E, F, I) phenotypes compared with the control worms (Fig. 5D, G). The results of the larval screens would suggest that the DPY-31 enzyme has the same crucial role during the development of both parasitic and free-living nematodes.

## 4 Discussion

Astacin metalloproteases play critical roles in cuticle synthesis and moulting in *C. elegans* (Hishida et al., 1996; Davis et al., 2004; Novelli et al., 2004; Suzuki et al., 2004) and parasitic nematodes (Stepek et al., 2010, 2011), with the astacin metalloprotease DPY-31 being essential for the procollagen cleavage step of cuticle synthesis (Novelli et al., 2006). In support of their importance in parasitic nematode development, related astacin-like metalloproteases have been independently identified as potential nematode vaccine candidates (Hotez et al., 2003; Barnard et al., 2012). Our previous analysis reported a high degree of functional conservation between DPY-31 from *C. elegans*, the ovine GI nematode *H. contortus*, and the human filarial nematode *Brugia malayi* (Stepek et al., 2010). This current study describes the detailed identification and characterisation of DPY-31 from the major GI nematode of sheep, *T. circumcincta*, and the identification of new inhibitors against this enzyme that may represent a starting point for development of novel chemotherapeutic agents.

Identity of 74% was noted between DPY-31 from *C. elegans* and *T. circumcincta*, compared with 70% between *C. elegans* and *H. contortus* (Stepek et al., 2010) and 89% between the proteins from *T. circumcincta* and *H. contortus*. This level of identity is consistent with the fact that all three species belong to nematode clade V. As discovered for the *dpy-31* genes from *H. contortus* and *B. malayi* (Stepek et al., 2010), *dpy-31* from *T. circumcincta* is much larger than the *C. elegans* gene; this is due to an increase in both the size and number of introns, and the completed genome of *H. contortus* confirms this to be a common feature in this parasite (Laing et al., 2013).

Using the parasite promoter to direct the expression of DPY-31 in *C. elegans* indicates that the expression of DPY-31 in *T. circumcincta* is analogous to that described for *B. malayi*, which was also determined using *C. elegans* as a surrogate system (Stepek et al., 2010); this expression was mainly observed in the pharyngeal gland cells and rectal epithelial cells throughout the life-cycle. This expression pattern overlaps with DPY-31 expression in *C. elegans*, although DPY-31 is also expressed in the hypodermis in the free-living species (Novelli et al., 2004). This partially shared expression pattern is consistent with a role for DPY-31 in nematode development since other proteases involved in cuticle synthesis of *C. elegans*, such as the astacin metalloproteases NAS-36 and NAS-37, are also present in the hypodermal cells, the pharyngeal and rectal epithelial cells (Davis et al., 2004; Suzuki et al., 2004). Thus, the expression pattern of DPY-31 in parasitic and free-living nematodes is consistent with a role in cuticle synthesis and this is further supported following *C. elegans* mutant rescue experiments. The *T. circumcincta dpy-31* gene was able to fully complement the temperature-sensitive phenotype of the *C. elegans dpy-31(e2770)* mutant by returning the wild-type form at the non-permissive temperature. RNAi knockdown of *T. circumcincta dpy-31* in this strain resulted in reversion to the mutant phenotype, supporting the hypothesis that *dpy-31* performs the same critical developmental role in parasitic nematodes as has been established in *C. elegans*. This provides compelling evidence to support the functional conservation of DPY-31 between parasitic nematodes and *C. elegans*.

The nematode DPY-31 enzymes share similarities with the vertebrate procollagen C-proteinase, BMP-1 (Novelli et al., 2004). BMP-1 is a vertebrate enzyme involved in bone formation through its ability to cleave the carboxyl terminal domains of procollagens (Li et al., 1996). In *C. elegans, dpy-31* genetic suppressor screens identified that this enzyme cleaved the short C-terminal domain of the essential cuticle collagen, SQT-3 (Novelli et al., 2006). This role was supported following *in vitro* cleavage experiments with recombinant DPY-31 from *B. malayi* and *H. contortus* (Stepek et al., 2010), indicating functional conservation of DPY-31 throughout the nematode phylum. This conserved nematode-specific function for DPY-31 makes this protease an ideal candidate as a target for new anti-nematode drugs. Following the discovery that hydroxamate-based compounds are effective inhibitors of homologous vertebrate procollagen C-proteinases (Ovens et al., 2000; Fish et al., 2007; Bailey et al., 2008), this current study established phenotypic and biochemical screens for five compounds with known matrix metalloproteinase inhibitory activity against *C. elegans*, *T. circumcincta* and *H. contortus* larvae and against recombinant *T. circumcincta* DPY-31 enzyme. Four of these compounds (1, 56, 25 and 26) were specifically developed to target procollagen C-proteinases (Fish et al., 2007) and two of these (25, 26) were the most potent inhibitors of recombinant DPY-31 from *T. circumcincta*. The effective concentrations of these inhibitors *in vitro* and *in vivo* were found to be in the micromolar range compared with the nanomolar range reported for the vertebrate enzymes (Fish et al., 2007). Nonetheless, the *in vivo* correlations between inhibitor phenotypes and the genetic mutants indicate that this is a promising starting point from which to develop more nematode-specific inhibitors. The phenotypic defects were comparable between *C. elegans* and a *C. elegans dpy-31* mutant rescued with the *T. circumcincta dpy-31* gene and more importantly, were consistently similar in *T. circumcincta* larvae. This suggests that the crucial role that DPY-31 plays in cuticle synthesis is conserved between these diverse free-living and parasitic nematodes, and is substantiated by the similar efficacy of these compounds against a second major GI nematode of sheep, *H. contortus*. The procollagen C-proteinase inhibitors 25 and 26 share an extra aromatic ring adjacent to the oxadiazole ring, a structure that is absent in the otherwise related compounds 1 and 56, and this may account for the enzyme specificity since additional pi-stacking interactions or H-bond interactions may be occurring through this structure. Thus, further investigations are warranted to establish whether members of this sub-class of hydroxamate-based inhibitors have potential promise as new drugs against nematode infections.

In conclusion, DPY-31 is a crucial procollagen C-proteinase in *C. elegans* and in parasitic nematodes that is involved in cuticle formation, most probably through the C-terminal cleavage of procollagens to form mature collagens. This present study, together with our previous report (Stepek et al., 2010), demonstrates that the function of DPY-31 is conserved throughout the nematode phylum and, hence, this enzyme class may represent an ideal target for future nematode control. This current study has demonstrated that a subset of hydroxamate-based compounds are effective inhibitors of DPY-31, and represent a potential starting point for the development of new drugs to combat important nematode infections.

## Figures and Tables

**Table 1:** Compounds with inhibitory activity against *Caenorhabditis elegans* L4-derived offspring (N2 and TP224), and *Teladorsagia circumcincta* L1, L3 and recombinant DPY-31 protein.

Compound	Structure^a^	Effect against *C. elegans* L4 progeny^c^		Effect against *T. circumcincta*^c^		Effective concentration against *T. circumcincta* rDPY-31^d^
		N2	TP224^b^	L1	L3	
Actinonin		SLO; STE; DPY/MLT	SLO; STE; DPY/MLT	SLO; DPY	DPY/MLT; Sick	50–100 μM (50 μM)
Compound 1		SLO; DPY	SLO; STE; DPY	ND	ND	x
Compound 56		SLO; STE; DPY	SLO; STE; DPY	ND	ND	x
Compound 26		SLO; LET; DPY	SLO; LET; DPY	ND	ND	50 μM (33 μM)
Compound 25		SLO; STE; DPY/MLT	SLO; STE; DPY/MLT	SLO	DPY/MLT; Sick	100 μM (82 μM)
1,10-phenanthroline		SLO; LET	SLO; LET	SLO	WT	100 μM
dH_2_O		WT	WT	WT	WT	x
5% Ethanol		WT	WT	WT	WT	x

a Hydroxamate functional group is highlighted in bold and grey.

b TP224, *dpy-31(e2770)* rescued with *T. circumcincta dpy-31.*

c Phenotypes observed were WT, wild-type; DPY, shorter, fatter body length compared with wild-type; MLT, old cuticle retained by new larval stage; STE, sterile or reduced number of progeny; SLO, immobile; LET, larval lethal (no further development due to death of larva); Sick, lethargic; ND, Not determined.

d LC_50_ values in brackets where it was possible to calculate those; x, no effect.

## Appendix A Supplementary data

Supplementary Table S1 Primers used in this study.
