# Supplementary material for: A highly conserved, inhibitable astacin metalloprotease from Teladorsagia circumcincta is required for cuticle formation and nematode development
Source: Int J Parasitol. 2015 Apr;45(5):345–55. doi: 10.1016/j.ijpara.2015.01.004 (PMC4406453; doi:10.1016/j.ijpara.2015.01.004)
Supplement: Supplementary Table S1 — Primers used in this study. [file mmc1.docx]

**Supplementary Table S1.** Primers used in this study.

| **Primer** | **Sequence** |
| --- | --- |
| dpy-315`1 | 5`- CAGATCCGATCCGATAAAC -3` |
| dpy-315`2 | 5`- CTTCAGGGTTCAGAAGTTC -3` |
| dpy-31a3` | 5`- CAGTGGAAAACCAAGTCTG -3` |
| Tc dpy-31SbfIF | 5`- GCGCCTGCAGGATGTCCCTATTACGCTGTAC -3` |
| Tc35aR | 5`- CGCGCGGCCGCTCACCGCACACAGCGAC -3` |
| Ce35pinF | 5`- CTTATGTGAACACTTGAC -3` |
| Ce35uinR | 5`- CTCGTTCAATCATACAC -3` |
| Tcdpy-31SIseqF | 5`- CAGATGATGTCGACAATG -3` |
| dpy-31F | 5`- GGGTTGGTTTGGCGGACTACTGATATGGTTGTAGT -3` |
| dpy-31(M)F | 5`- GGGCGACTACTGATATGGTTGTATC -3` |
| dpy-31R | 5`- TTAGCTATGGTTATATAAAG -3` |
| dpy-31R2 | 5`- CTCCAGGAATTCCAGTTAC -3` |
| Tc dpy-31repF2 | 5`- GCGGTCGACGTACAAATGGGAGAG -3` |
| Tc dpy-31repR2 | 5`- CGCCCCGGGTAGGGACATTGCGGTCGTCAG -3` |
| Reporterseq | 5`- GACAACTCCAGTGAAAAG -3` |
| M13Rev(-29) | 5`- CAGGAAACAGCTATGACC -3` |
